# Utilization of Automated Keyword Search to Identify E-Scooter Injuries in the Emergency Department

**DOI:** 10.7759/cureus.19539

**Published:** 2021-11-13

**Authors:** Ali Pourmand, Keith S Boniface, Katherine Douglass, Colton Hood, Sarah E Frasure, Jeremy Barnett, Kunj Bhatt, Neal Sikka

**Affiliations:** 1 Emergency Medicine, George Washington University School of Medicine and Health Sciences, Washington, DC, USA

**Keywords:** traumatic injuries, python text search, emergency department, scooter, natural language processing

## Abstract

Background and objective

Accurate identification and categorization of injuries from medical records can be challenging, yet it is important for injury epidemiology and prevention efforts. Coding systems such as the International Classification of Diseases (ICD) have well-known limitations. Utilizing computer-based techniques such as natural language processing (NLP) can help augment the identification and categorization of diseases in electronic health records. We used a Python program to search the text to identify cases of scooter injuries that presented to our emergency department (ED).

Materials and methods

This retrospective chart review was conducted between March 2017 and June 2019 in a single, urban academic ED with approximately 80,000 annual visits. The physician documentation was stored as combined PDF files by date. A Python program was developed to search the text from 186,987 encounters to find the string “scoot” and to extract the 100 characters before and after the phrase to facilitate a manual review of this subset of charts.

Results

A total of 890 charts were identified using the Python program, of which 235 (26.4%) were confirmed as e-scooter cases. Patients had an average age of 36 years and 53% were male. In 81.7% of cases, the patients reported a fall from the scooter and only 1.7% reported wearing a helmet during the event. The most commonly injured body areas were the upper extremity (57.9%), head (42.1%), and lower extremity (36.2%). The most frequently consulted specialists were orthopedic and trauma surgeons with 28% of cases requiring a consult. In our population, 9.4% of patients required admission to the hospital.

Conclusions

The number of results and data returned by the Python program was easy to manage and made it easier to identify charts for abstraction. The charts obtained allowed us to understand the nature and demographics of e-scooter injuries in our ED. E-scooters continue to be a popular mode of transportation, and understanding injury patterns related to them may inform and guide opportunities for policy and prevention.

## Introduction

Dockless e-scooters saw tremendous growth in 2017 and rapidly gained popularity as a convenient and environmentally-friendly form of transportation [1]. Several reports of injuries related to e-scooters followed soon after as riders began presenting to emergency departments (EDs) and acute care settings around the country [2,3]. A clear understanding of injury epidemiology is important to inform policy decisions, including aspects such as speed regulations, safe locations for riding, and safety practices for riders. Timely identification of emerging injuries and illnesses is an essential component of injury surveillance. Prevention programs may be delayed due to poor and fragmented data collected from clinical systems, such as electronic health records [4]. The International Classification of Diseases (ICD) was developed by the World Health Organization (WHO) and has been adopted by countries to standardize the documentation of clinical diagnoses for a variety of purposes. This classification system is used to identify the trends, causes, and outcomes of medical cases, but the use of ICD codes as a primary source of data collection can lead to gaps in information, particularly related to the mechanism of injury [5]. Under extraordinary circumstances, such as in response to the ongoing coronavirus disease 2019 (COVID-19) pandemic, ICD-10 codes have been quickly updated and adopted; however, this is not typical [6]. For example, for injuries related to e-scooters, the ICD-10-CM V00.8441A “Fall from standing electric scooter, initial encounter” was not added until October 2020 [7]. Potentially dangerous injuries could be inconsistently reported by the emergency medicine community through the improper use of ICD-10 codes [8] and both the underutilization of as well as the erroneous use of ICD-10 codes poses a challenge to attaining a comprehensive understanding of specific domains such as work-related injuries in the ED [9].

Natural language processing (NLP) refers to the field that combines computer science, artificial intelligence, and linguistics to enable computers to understand or even create human languages [10]. Clinicians documenting patient care in the electronic health record generate significant amounts of free text, and NLP can be used to bring meaning and structure for further analysis [11]. Processing free text documentation has been used in the past to accurately recognize simple wording in medical language to identify potentially underreported diseases when analyzed only by ICD-10 classification [12]. In combination with ICD codes, it can help identify uncommon but serious diseases [13] and free text can be searched to identify conditions independent of the ICD code [14].

Like other EDs, our urban tertiary care center witnessed increasing cases of injuries related to e-scooters soon after their introduction within the community. Being aware of the limitations and challenges of extracting injury information from our electronic health record, we undertook this study to better understand the epidemiology of e-scooter-related injuries. Our primary objective was to identify e-scooter injuries by using a Python computer program to search the text of our ED physician documentation. Our secondary objective was to characterize the patient demographics and injury patterns associated with e-scooter use that result in ED visits.

This article was previously presented as an abstract at the ACEP Research Forum, October 26-29, 2020 (Douglass K, Sikka N, Boniface K, Bhatt K, McCarville P, Pourmand A. Epidemiological Analysis of E-Scooter Injuries among Patients Presenting to the Emergency Department. ACEP Research Forum; October 26-29, 2020).

## Materials and methods

This retrospective chart review was conducted between March 2017 and June 2019 in a single, urban academic ED with approximately 80,000 annual visits. The physician documentation was stored as combined PDF files by date. A Python program was used to prepare and search the text from 186,987 encounters to identify charts. First, the text was converted to lower case, and scooter injuries were identified using the standard library string method to locate occurrences of “scoot” and to extract the 100 characters before and after this location. This string along with the unique identifier of its encounter was written into a comma-separated value (CSV) file to facilitate a manual review of these identified charts. An error file was generated to list any files that could not be opened or processed. We included all patients identified as presenting to the ED with an e-scooter-related injury.

Charts identified in the keyword search were reviewed by research assistants against our inclusion criteria. Research assistants were trained in data abstraction, and progress was monitored through regular meetings. A standard data abstraction form was developed by the research team including patient-centered epidemiologic data, injuries sustained, treatments rendered, and circumstances surrounding the injury. No personally identifiable information was included. The form was pilot tested, finalized, and entered into a Research Electronic Data Capture (REDCap) database for secure collection and storage. Study data were collected and managed using REDCap electronic data capture tools hosted at (BLINDED). REDCap is a secure, web-based software platform designed to support data capture for research studies, providing (1) an intuitive interface for validated data capture, (2) audit trails for tracking data manipulation and export procedures, (3) automated export procedures for seamless data downloads to common statistical packages, and (4) procedures for data integration and interoperability with external sources [15,16].

Of note, 10% of charts were randomly selected for abstraction by a second blinded researcher to assess inter-observer reliability. Data were analyzed using descriptive and summary statistics. The study was reviewed and deemed exempt from approval by our institutional review board.

## Results

A total of 186,987 electronic charts comprising 1,153,882 pages were searched. The search was completed in 9.26 hours. It took an average of 0.029 seconds to search each page and 0.178 seconds to search each chart. The search identified 890 charts that contained the string “scoot”. A total of 235 (26.4%) e-scooter cases were identified upon manual review of the search results. The error file contained no entries.

Table 1 presents the characteristics of the patients injured in e-scooter crashes. Because our hospital catchment area includes Washington DC, Maryland, and Virginia, the majority of patients resided in one of these jurisdictions. In 81.7% of injuries, the patient reported a fall from the scooter, and only 1.7% reported wearing a helmet during the event. The most commonly injured body areas were the upper extremity (57.9%), head (42.1%), and lower extremity (36.2%) with the injuries described as abrasion (82.5%), fracture (39.1%), and laceration (31.5%). X-ray and CT scans were performed on 69.4% and 34.5% of patients respectively. Specialists, most commonly orthopedic and trauma surgeons, were consulted in 28% of cases; 9.4% of patients required hospital admission.

**Table 1:** Patient characteristics (n=235) IQR: interquartile range

Characteristics	Values
Patient sex/gender, n (%)	
Male	124 (52.8%)
Female	111 (47.2%)
Patient age, years	
Mean	36.02
Median (IQR)	31 (23)
Range	7–89
State of residence, n (%)	
Washington, DC	116 (49.4%)
Maryland	14 (6.0%)
Virginia	22 (9.4%)
Other	83 (35.3%)
Position on the scooter, n (%)	
Riding alone on the scooter	206 (87.7%)
Riding double, in the front	5 (2.1%)
Riding double, in the back	1 (0.4%)
Injured by someone riding on a scooter	4 (1.7%)
Otherwise injured related to a scooter	7 (3.0%)
Not recorded	12 (5.1%)
Location of the scooter at the time of the incident, n (%)	
Street	107 (45.5%)
Sidewalk	19 (8.1%)
Bike lane	1 (0.4%)
Not recorded	108 (46.0%)
Circumstances of the incident, n (%)	
Collision	35 (14.9%)
Related to uneven surface	35 (14.9%)
Lost control	32 (13.6%)
Fell off the device	192 (81.7%)
Other	16 (6.8%)
Not recorded	6 (2.6%)
Reported alcohol or drug usage, n (%)	
Alcohol	19 (8.1%)
Drug use	1 (0.4%)

Table 2 describes the injury characteristics.

**Table 2:** Injury characteristics *Of note, some patients fit into multiple categories, and hence percentages may add up to >100% and numbers may exceed 235 CT: computed tomography; ED: emergency department; ENT: ears, nose, and throat; ICU: intensive care unit; MRI: magnetic resonance imaging

Characteristics	Values
Area of injury/physical exam finding, n (%)	
Head	99 (42.1%)
Upper extremity	136 (57.9%)
Lower extremity	85 (36.2%)
Trunk	13 (5.5%)
Other	9 (3.8%)
Type of injury*, n (%)	
Abrasion	123 (52.3%)
Contusion	55 (23.4%)
Concussion	13 (5.5%)
Laceration	74 (31.5%)
Fracture	92 (39.1%)
Dislocation	4 (1.7%)
Other	32 (13.6%)
Imaging procedures in ED*, n (%)	
X-ray	163 (69.4%)
CT	81 (34.5%)
MRI	1 (0.4%)
Ultrasound	6 (2.6%)
None	32 (13.6%)
Type of specialty consultation*, n (%)	
Orthopedic surgery	36 (15.3%)
Neurosurgery	8 (3.4%)
Trauma surgery	21 (8.9%)
ENT	12 (5.1%)
Ophthalmology	5 (2.1%)
Neurology	2 (0.9%)
Plastic surgery	1 (0.4%)
Patient disposition, n (%)	
Home	215 (91.5%)
Admitted to floor	17 (7.2%)
Admitted to ICU	3 (1.3%)

Of the 235 patient charts reviewed, 185 (78.7%) included an external cause code related to the mechanism of injury (“e-code”). Of those charts with an e-code, 53% of those codes included the word “scooter” (Table 3). The other 47% included various mechanisms of injury, many related to falls or motor vehicle collisions.

**Table 3:** Mechanism of injury codes ICD: International Classification of Diseases

ICD-10 code	Number of patients (percentage)
V00141A (fall from scooter)	27 (14.6%)
V00831A (fall from motorized mobility scooter)	50 (27.0%)
WO51XXA (fall from non-moving, non-motorized scooter)	10 (5.4%)
V00832A (motorized mobility scooter colliding with stationary object)	2 (1.1%)
W052XXA (fall from non-moving motorized mobility scooter)	5 (2.7%)
V00148A [other scooter (non-motorized) accident]	2 (1.1%)
V00142A [scooter (non-motorized) colliding with stationary object]	1 (0.5%)
V00838A (other accident with motorized mobility scooter)	1 (0.5%)
Non-scooter codes	87 (47.0%)

## Discussion

Real-time identification of emerging patterns of disease is an ongoing challenge encountered in injury research and prevention and other emerging diseases. Reliance on ICD-10 coding to incorporate both mechanism of injury and actual injury patterns into electronic medical records offers an incomplete solution, although textual components of the chart will often include important factors omitted in the codes. As described in this study, only 52% of the reviewed charts had ICD-10 codes including the word scooter, and none of the scooter-related codes were appropriate for the new e-scooter device. The 2021 version of ICD-10 includes an extensive array of updated codes related to e-scooters [17], although clinician coding will always have some form of expected deficiency [18]. This study described a pilot effort to identify incidents of e-scooter injury using text searching to improve and expedite the determination of the epidemiologic profile of injuries related to an evolving transportation technology.

Meanwhile, e-scooters have continued to grow in popularity [19] due to their environmental benefits while continuing to be a source of injuries worldwide. Urban centers may see different patterns of injury depending on various characteristics such as road infrastructure, availability of bike lanes, topography, and level of tourism. For example, in our study, over 35% of patients with scooter-related injuries reside outside our local area. A better understanding of injury patterns will help to improve policymaking and prevention strategies. In our study, 45.5% of scooter crashes occurred on the street, which may influence future policy changes in trafficking dynamics with e-scooters in injury prevention. We also found that 8.1% of our e-scooter riders reported alcohol use while operating the e-scooter, which may increase the likelihood of crashes as well as the number and severity of injuries. Our data detail a relatively low acuity injury pattern, as 91.5% of patients were discharged home from the ED. However, the vast majority of patients (86.4%) required imaging studies of some kind, and 28% required specialty consultation. More detailed information regarding rider factors, such as experience in riding and familiarity with routes, and environmental factors such as dangerous intersections will be important to fully understand opportunities for injury prevention.

The review of the charts was conducted at a single center, limiting the population catchment, which may make the generalization of the results difficult. Epidemiological data may be more difficult to find based on the charting style of different medical professionals. We did not identify cases using ICD-10 codes but used a search string and surrounding characters to locate charts; further research will be needed to evaluate the effectiveness of the tool in retrospective chart reviews in the ED. Multi-center studies in the future will be effective in providing deeper insights into the injury pattern, population, and ED treatment patterns.

## Conclusions

The Python program used in this study was able to identify charts related to scooter injuries based on the search for the string “scoot” in the ED physician documentation text. The search returned a manageable number of charts, in addition to 100 words surrounding the location of the target string to enable the rapid identification and abstraction of e-scooter injuries. Data collection from these charts provided an understanding of the demographics and circumstances of e-scooter-related injuries presenting to our ED in the District of Columbia. E-scooters continue to be a popular mode of travel in urban centers as well as a major source of injuries that present to the ED. Future research should combine analysis of the text with ICD-10 codes to maximize the identification of potential injuries. Lastly, our study method combined with ICD search results should help us identify and categorize injury documentation, thereby allowing us to create a training set for the future development of more advanced injury identification using machine learning techniques such as NLP.
